# Gene Expression Profiling of Pancreatic Ductal Adenocarcinoma Arising From Intraductal Papillary Mucinous Neoplasms of the Pancreas

**DOI:** 10.1002/cam4.70499

**Published:** 2024-12-11

**Authors:** William A. Ziaziaris, Christopher S. H. Lim, Loretta Sioson, Anthony J. Gill, Jaswinder S. Samra, Sumit Sahni, Anubhav Mittal

**Affiliations:** ^1^ Department of Upper Gastrointestinal Surgery Royal North Shore Hospital Sydney Australia; ^2^ Faculty of Medical and Health The University of Sydney Sydney Australia; ^3^ NSW Health Pathology, Department of Anatomical Pathology Royal North Shore Hospital Sydney Australia; ^4^ Australian Pancreatic Centre Sydney Australia; ^5^ Kolling Institute of Medical Research The University of Sydney Sydney Australia; ^6^ School of Medicine The University of Notre Dame Sydney Australia

**Keywords:** gene expression analysis, intraductal pancreatic mucinous neoplasm (IPMN), molecular biomarkers, pancreatic cysts, pancreatic ductal adenocarcinoma, pancreatic neoplasms

## Abstract

**Introduction:**

Intraductal papillary mucinous neoplasms (IPMNs) are diverse premalignant tumors of the pancreas. They progress stepwise from adenoma to carcinoma and offer an opportunity for intervention prior to malignant transformation into pancreatic ductal adenocarcinoma (PDAC). The current study aimed to identify differentially expressed genes (DEGs) in invasive PDAC‐associated IPMN vs. noninvasive IPMN to understand the potential molecular changes involved in malignant transformation of IPMN into PDAC.

**Materials and Methods:**

Archived tissue and data from 12 patients with histologically proven invasive PDAC arising from IPMN specimens were assessed. Gene expression analysis was performed on RNA extracted from macro‐dissected tissue specimens using the NanoString nCounter PanCancer Progression assay. Statistical and pathway analysis was performed using SPSS v28 and Ingenuity Pathway Analysis, respectively.

**Results:**

A total of 159 genes had significantly (*p* < 0.05, *q* < 0.05) different expression in PDAC arising from IPMN compared with that from IPMN alone (91 overexpressed and 68 underexpressed). Interestingly, 14 of top 10 over‐ and underexpressed genes were predicted to translate secretory proteins, with SignalP scores approaching 1. A number of differential canonical pathways (e.g., LXR/RXR activation pathway, glycolysis I gluconeogenesis I, and hepatic fibrosis) and potential upstream regulators (e.g., TGFB1, THBS2, etc.) were also identified.

**Conclusion:**

A differential gene expression profile between PDAC arising from IPMN and IPMN alone was identified. Pathway analysis identified potential mechanisms involved in malignant transformation of IPMN to PDAC.

## Graphical abstract

Intraductal papillary mucinous neoplasms (IPMNs) are diverse premalignant tumors of the pancreas. They progress stepwise from adenoma to carcinoma and offer an opportunity for intervention prior to malignant transformation into pancreatic ductal adenocarcinoma (PDAC). In this study, we identified differentially expressed genes (DEGs) in invasive PDAC‐associated IPMN versus noninvasive IPMN to establish potential molecular biomarkers for risk stratification.

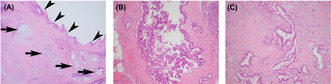

## 1 Introduction

Intraductal papillary mucinous neoplasms (IPMNs) of the pancreas are morphologically and histologically diverse premalignant tumors of the exocrine pancreas [1]. They are classified into four subtypes including gastric, intestinal, pancreaticobiliary, and oncocytic; with the gastric subtype being the most commonly diagnosed upon resection [1, 2].

IPMN progresses in a stepwise fashion over time from adenoma to carcinoma [3]. Although invasive carcinoma of the pancreas has a poor prognosis, premalignant IPMNs offer an opportunity for intervention prior to malignant transformation. Currently, international consensus guidelines such as the Fukuoka, European, and American Gastroenterological Association (AGA) guidelines use a mixture of clinical and radiological features to risk‐stratify IPMNs that are suspicious for malignancy and thus warrant resection [4]. These guidelines are varied in their recommendations for resection. Although high‐risk stigmata such as abdominal symptoms, size of lesion, or the presence of main pancreatic duct dilatation or intramural nodules have been shown to predict malignancy, these are imperfect, with a reported diagnostic accuracy of about 75% [5, 6]. With the morbidity of partial pancreatectomy approaching 30%–40%, differentiating high‐ versus low‐risk IPMN is paramount to avoid unnecessary morbidity and mortality. Conversely, resection of high‐risk IPMN is important in preventing the inevitable and devastating diagnosis of pancreatic ductal adenocarcinoma (PDAC) [7].

Studies have attempted to identify molecular biomarkers that could be used in conjunction with radiological and clinical features to predict the malignant potential of these tumors more accurately [6, 8, 9, 10, 11, 12, 13]. This exploratory study aims to build on previous works by identifying differentially expressed genes (DEGs) in invasive PDAC arising from IPMN versus IPMN alone. The potential pathways involved in the progression of IPMN to invasive carcinoma were also identified.

## 2 Materials and Methods

### 2.1 Ethics Approval

Ethical approval was obtained from the Northern Sydney Local Health District Human Research Ethics Council (NSLHD HREC, reference 2019/ETH0863). A waiver of consent was obtained from NSLHD HREC to use archived tissue blocks under NSW Human Tissue Act 1983.

### 2.2 Study Design

Patients who had partial or total pancreatectomy with histologically proven PDAC arising from IPMN from 2016 to 2021 with archived formalin‐fixed and paraffin‐embedded tissue blocks available through the Department Anatomical Pathology, Royal North Shore Hospital (Sydney, Australia), were included. Tissue blocks with tumor cellularity less than 20% were excluded.

**FIGURE 1:**
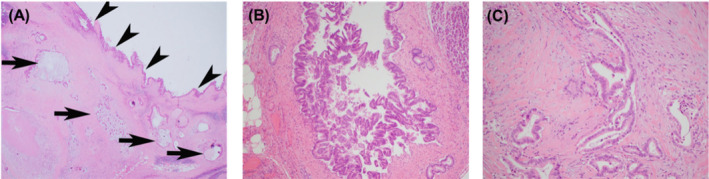
Representative images of specimens. (A) Pancreatic ductal adenocarcinoma (PDAC) arising from the intraductal papillary mucinous neoplasm (IPMN). IPMN (arrowheads) is composed of a villiform proliferation of atypical epithelial cells lining a dilated duct. PDAC (arrows) arising from the IPMN demonstrates greater cytological architecture and an infiltrative growth pattern (H&E, original magnification 20×). (B) IPMN: The tumor demonstrates a villiform architecture growing into the duct system. There is no invasive growth (H&E, original magnification 100×). (C) Adenocarcinoma. The malignant cells demonstrate a well‐developed acinar architecture with a clearly invasive growth pattern, so that the malignant cells are separated by desmoplastic stroma (H&E original magnification 200×).

Tissue sections were macrodissected for both the PDAC component and IPMN component (without carcinoma), thereby serving as their own controls. Twelve patients each with two samples (PDAC and IPMN components) were included in the study (Figure 1). The DEGs of these two groups were then compared.

### 2.3 Gene Expression Analysis

Total RNA was extracted using Qiagen AllPrep DNA/RNA FFPE Kit (Cat# 80234; Qiagen, Hilden, Germany) following the manufacturer's protocol, and then quantified and assessed for fragmentation using Bioanalyzer Nano Kit (Agilent). Specimens then underwent RNA expression analysis of 800 genes using the nCounter PanCancer Progression Panel (NanoString Technologies Inc., Seattle, WA, USA, 2022) with additional 30 genes added to the panel (Table S1) using manufacturer's protocol. Gene expression data were imported and quality control performed using nSolver 4.0 software (Nanostring Technologies Inc).

### 2.4 Data Analysis

Gene expression data were normalized using 30 house‐keeping genes on nSolver 4.0 software (NanoString Technologies Inc). Statistical analysis was performed using SPSS v28 (IBM, Armonk, NY, USA). Data were assessed for normality with Shapiro–Wilk testing and comparison between gene expression in PDAC arising from IPMN and IPMN tissue samples was performed with Mann–Whitney‐*U* test. For all analyses, *p* value < 0.05 were accepted as statistically significant. False discovery rate (FDR) correction was then performed using the Benjamini and Hochberg method with *q* value < 0.05 deemed significant. Signal peptide analysis was performed to identify potential genes with secretory protein products using SignalP 6.0 (Technical University of Denmark) [14].

Gene expression that differed statistically between samples of IPMN and PDAC arising from IPMN were then compared with fold‐change calculations. Pathways analysis was then performed on statistically significant genes with a fold‐change cutoff of 1.5 using Ingenuity Pathways Analysis (IPA; Qiagen Bioinformatics, Redwood City, CA).

## 3 Results

### 3.1 Population Demographics

A total of 12 patients (7 males and 5 females) with concurrent PDAC arising from IPMN were included in this study. The mean age at resection was 72 years. Five patients (42%) underwent neoadjuvant chemotherapy prior to surgical resection. Patient demographics are summarized in Table 1.

**TABLE 1:** Patient demographics demonstrating heterogeneity in patient population.

Characteristic	*N* (%)
Sex	
Male	7 (58%)
Female	5 (42%)
Age	72 (SD 8.8)
Neoadjuvant chemotherapy	5 (42%)
Surgery type	
Pancreaticoduodenectomy	7 (58%)
Distal pancreatectomy	4 (33%)
Total pancreatectomy	1 (8%)
Histopathology	
PDAC	
Poorly differentiated	3
Moderately differentiated	8
Well differentiated	1
IPMN	
Low‐grade dysplasia	4
High‐grade dysplasia	8

### 3.2 Gene Expression Analysis

Of the 800 genes in the nCounter assay, the expression of 177 was significantly different between PDAC‐associated IPMN and IPMN samples (*p* < 0.05). After FDR correction, 159 genes were significant (*q* < 0.05; Table S2). The top 10 over‐ and underexpressed genes in PDAC arising from IPMN are reported in Table 2.

**TABLE 2:** Top 10 over‐ and underexpressed genes between invasive PDAC‐associated IPMN and non‐invasive IPMN.

Gene	Fold change	*p*	Signal*P* score	Number of up‐ or downregulated genes
**Overexpressed genes**				
S100A2	4.56947261	0.023	0	75% (9/12)
MUC16	3.70439779	0.004	0.0001	67% (8/12)
CEACAM6	3.43760502	0.008	0.9988	75% (9/12)
ELK3	2.93547021	< 0.001	0	100% (12/12)
CLDN4	2.84362215	0.002	0.0537	100% (12/12)
CEACAM1	2.76286874	0.014	0.9997	83% (10/12)
IL1RN	2.70557709	0.028	0.9997	83% (10/12)
PLAUR	2.61969215	0.014	0.9998	83% (10/12)
KRT19	2.57952602	0.024	0	75% (9/12)
S100A14	2.57738883	0.035	0	83% (10/12)
**Underexpressed genes**				
*ALB*	0.03682218	0.026	0.9997	58% (7/12)
*PTX3*	0.18664636	0.011	0.9998	92% (11/12)
*CHRDL1*	0.23396379	< 0.001	0.9997	92% (11/12)
*CLU*	0.29541266	< 0.001	0	92% (11/12)
*OGN*	0.33154706	0.002	0.9998	92% (11/12)
*TFPI2*	0.3432229	0.023	0.9998	75% (9/12)
*ITIH4*	0.36572222	0.013	0.9998	100% (12/12)
*SLC44A4*	0.3790737	0.002	0	75% (9/12)
*STAB1*	0.38187688	0.002	0.9998	92% (11/12)
*ADAM28*	0.38347491	0.011	0.9985	92% (11/12)

### 3.3 Signal Peptide Analysis

Of the 159 genes that differed significantly between components, 61 had SignalP scores > 0.95, demonstrating high likelihood of signal peptide presence (Table S2). Of the top 10 most over‐ and underexpressed genes, *CEACAM6*, *CEACAM1*, *IL1RN*, *PLAUR*, *ALB*, *PTX3*, *CHRDL1*, *OGN*, *TFPI2*, *ITIH4*, *STAB1* and *ADAM28* (12 of 20) had Signal*P* scores approaching 1 (Table 2).

### 3.4 Pathways Analysis

**FIGURE 2:**
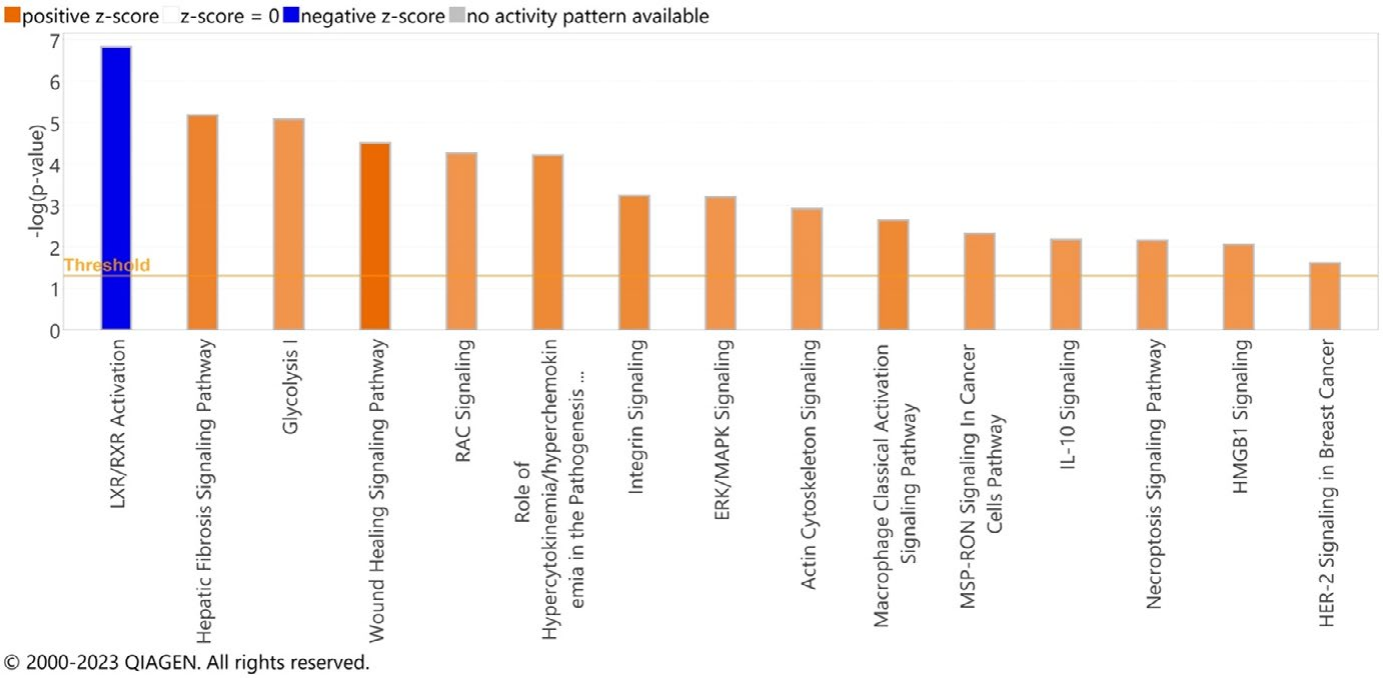
Canonical pathway analysis demonstrating 15 significant pathways with a *z*‐score ≥ 2 (orange) and ≤ 2 (blue) for up‐ and downregulated pathways, respectively.

On the basis of differences in gene expression, a pathways analysis was performed using Ingenuity Pathways Analysis (Figure S1). A number of significantly upregulated canonical pathways were observed between invasive PDAC‐associated IPMN compared to non‐invasive IPMN (Figure 2), namely, actin cytoskeleton signaling I (*z*‐score = 2.00, −log(*p*) = 2.92), EPK/MAPK (*z*‐score = 2.00, −log(*p*) = 3.20), glycolysis I (*z*‐score = 2.00, −log(*p*) = 5.08), hepatic fibrosis (*z*‐score = 2.33, −log(*p*) = 5.18), HER2 (*z*‐score = 2.00, −log(*p*) = 1.61), HMGB1 (*z*‐score = 2.00, −log(*p*) = 2.05), IL‐10 signaling (*z*‐score = 2.00, −log(*p*) = 2.18), integrin (*z*‐score = 2.24, −log(*p*) = 3.23), macrophage classical activation (*z*‐score = 2.24, −log(*p*) = 2.64), MSP‐RON (*z*‐score = 2.00, −log(*p*) = 2.32), necroptosis (*z*‐score = 2.00, −log(*p*) = 2.16), RAC (*z*‐score = 2.00, −log(*p*) = 4.26), hypercytokinaemia/chemokinaemia (*z*‐score = 2.24, −log(*p*) = 4.21), and wound healing (*z*‐score = 2.83, −log(*p*) = 4.50) signaling pathways. In contrast, only one significantly downregulated pathway was observed, that is, LXR/RXR activation pathway (*z*‐score = −2.12, −log(*p*) = 6.82).

Fifty significant upstream regulators were also identified using Ingenuity Pathways Analysis (Table S3). These included transcription regulators, enzymes, cytokines, growth factors, transmembrane receptors, and transporters. Of these, *TGFB1*, *STAT1*, and *THBS2* were included in our gene assay, with activation *z*‐scores 4.641, 2.41, and 2.0; experimental fold changes 1.687, 1.908, and 2.07; *p* value of overlap = 1.65E‐31, 2.00E‐4, and 4.06E‐7; *q* = 0.003, 0.004, and 0.02, respectively. *STAT1* was involved in 5 of 14 upregulated pathways including EPK/MAPK, IL10, necroptosis, hypercytokinaemia/chemokinaemia, macrophage classical activation, and wound healing signaling pathways. TGFB1 was involved in four upregulated signaling pathways including hepatic fibrosis, HMGB1, macrophage classical activation, and wound healing. THBS2 was not involved in any significantly down‐ or upregulated pathways.

## 4 Discussion

The key to managing pancreatic cystic neoplasms such as IPMN is to understand the key molecular alternations that lead to their malignant transformation. We have identified multiple genes, pathways, and upstream regulators that are differentially expressed in PDAC arising from IPMN. However, as with all DEG studies, these results should be interpreted with caution as many genes have both oncogenic and tumor suppressor functions depending on both the type of malignancy and the stage of disease.

We demonstrated that a number of genes known to predict poor survival, metastatic disease phenotype, and chemo‐resistance in PDAC were also upregulated in invasive PDAC arising from IPMN. *CEACAM6*, *S100A2*, and *MUC16* genes are poor prognostic biomarkers and are associated with increased risk of lymph node and distal metastasis in PDAC [13, 15, 16, 17, 18, 19, 20, 21, 22]. CEACAM6 and *SLC2A1* may also play a role in IPMN tumor progression, with increased expression of these genes in high‐grade versus low‐ and intermediate‐grade IPMNs being previously observed [19, 22, 23, 24].

*ALB*, coding for the plasma protein albumin, is well known to be downregulated in PDAC although this may be more of an association rather than a causative factor [25, 26]. *ITIH4* is more controversial, with some studies demonstrating up‐ and downregulation in various cancers and stages of disease [27, 28, 29]. Here, we found that both *ALB* and *ITIH4* are reduced in PDAC‐associated IPMN. Both genes are involved in the one downregulated pathway seen in the current study, the LXR/RXR pathway, which functions in the cytotoxic T‐cell response to neoplasia [30].

We found 14 upregulated pathways in PDAC arising from IPMN compared with those from IPMN alone with a variety of functions. These include regulation of cell death and metabolism, DNA repair, extracellular matrix deposition, immune response, and tumor cell migration. Pathways involving extracellular matrix regulation such as the hepatic fibrosis and wound healing signaling pathways, have been previously implicated in PDAC and other cancers, creating a microenvironment that sustains PDAC cell growth [31, 32]. Progressive fibrosis and accumulation of stromal tissue is also a characteristic of premalignant intraepithelial lesions of the pancreas [33].

*IL1RN*, *STAT1*, and *TGFB1* are key genes involved in these pathways and were significantly upregulated. In PDAC, the IL‐1 family has been implicated in altering the tumor immune microenvironment, with higher tumor expression of *IL1RN* associated with poorer survival [34]. There are currently several drugs targeting the IL‐1 axis undergoing clinical trials, although their effects on the tumor immune microenvironment are as yet unknown [35, 36].

*TGFB1* signaling in pancreatic cancer is complex, having both an anti‐ and pro‐tumorigenic function. As such, the utility of TGFB1 inhibitors remains uncertain. Hezel et al. showed TGFB1 blockade‐accelerated early and late‐stage pancreatic cancer, whereas others have demonstrated tumor suppressor activity in early stages of PDAC and tumor promotion in later stages of the disease [37, 38]. TGFB1 also plays a significant role in pancreatic cancer chemoresistance, with activation of pancreatic stellate cells leading to excessive ECM deposition and fibrosis impairing drug delivery to cells [39, 40, 41, 42]. Research into TGFB1 axis modulation in pancreatic cancer is expanding. One single‐arm Phase 2 trial in patients with locally advanced PDAC demonstrated that the addition of the TGFB1 inhibitor losartan to neoadjuvant FOLFIRINOX and chemoradiotherapy decreased circulating TGFB1 and was associated with an R0 resection rate of 61% [43]. Additional drugs targeting TGFB1 mRNA and TGFB2 are also currently being evaluated [44, 45].

Signal transducers and activators of transcription 1 (STAT1) has been identified as a prognostic biomarker in PDAC and other solid cancers [46, 47]. Like TGFB1, STAT1 likely has both an anti‐ and pro‐tumorigenic effect [46, 47, 48, 49, 50, 51]. In one study, STAT1 activation led to DcR3‐mediated increased proliferation and invasion of pancreatic cells [48]. Others have demonstrated improved gemcitabine sensitivity with interferon γ‐mediated phosphorylation of STAT1 causing downregulation of the FOXM1 oncoprotein [47]. Although in vitro and in vivo data suggest a potential role for interferons in pancreatic cancer, clinical trial data remain lacking [52].

Several recent studies have identified key upregulated gene markers in gastric, pancreatobiliary, and intestinal IPMN subtypes [23, 53, 54]. *NKX6‐2* encodes for a homeobox domain‐containing transcription factor and is overexpressed in gastric‐subtype IPMNs [23, 54]. Similarly, *SPDEF* expression is elevated in intestinal‐subtype IPMNs and has an inverse correlation with *NKX6‐2* [54]. For this reason, *NKX6‐2* and *SPDEF* have emerged as specific gene markers of gastric and intestinal IPMN subtypes and may help indicate degree of dysplasia in IPMN [54]. Of these, SPDEF was predicted to be a significantly inhibited upstream regulator in our study (Table S3). In comparison, HOXB3 expression decreases with dysplasia, is absent in IPMNs with high‐grade dysplasia, and is therefore a potential marker of low‐grade IPMN [54]. In the present study, we demonstrated upregulation of HOXB3 in invasive carcinoma compared to associated IPMN (Table S2). *HOXB3* therefore may be a specific marker for high‐grade dysplasia only, the expression of which, if biphasic, could be increased on progression to invasive carcinoma. Iyer et al. [53] found 30 genes overexpressed and eight underexpressed in high‐grade versus low‐grade IPMNs, respectively. Of these high‐risk genes, *PLAUR* (overexpressed) and *CLU* (underexpressed) also demonstrated significant relationships in our study.

In addition to genetic markers, serum biomarkers are increasingly being utilized to help inform treatment decisions, both surgical and oncological in various malignancies [13, 55]. Signal peptides are short amino acid sequences that are found on proteins destined for secretion out of cells or for integration within the cell membrane [56, 57]. In contrast to the relatively inaccessible and expensive genetic sequencing of tumor cells, blood‐based biomarker assessment of proteins with high Signal*P* scores using ELISA may offer a noninvasive, cheaper alternative. Here, we demonstrate a number of DEGs in PDAC arising from IPMN with high Signal*P* scores allowing for serum measurement.

There are several limitations of this study. An important issue that could have resulted in confounding results is concomitant presence of a low‐grade IPMN with invasive carcinoma in tissue, rather than a genetically linked IPMN progressing to PDAC. To minimize this pitfall, an experienced pathologist chose the cases where PDAC appeared to arise in direct continuity with the IPMN. Moreover, the small cohort of 12 patients limits the predictive value of identified DEGs. Further, as PDAC‐associated IPMN and IPMN specimens were taken from the same patient, survival analysis could not be performed. There was also heterogeneity in the patient population, with 5 of 12 receiving neoadjuvant chemotherapy and variability in the grade of IPMN dysplasia and PDAC differentiation on histopathology. Future studies with larger cohorts encompassing a variety of patient presentations and histopathological subclasses of IPMN are therefore required to further validate the presented findings.

## 5 Conclusion

This exploratory study has identified molecular alterations involved in malignant transformation of IPMN. These findings require further analysis with larger cohorts including high‐ and low‐grade IPMN specimens to help better distinguish high‐ from low‐risk IPMNs and targetable pathways for PDAC treatment. Seven key genes, namely, *CEACAM1*, *CEACAM6*, *IL1RN*, *PLAUR*, *STAT1*, *TGFB1*, and *THBS2*, offer clinicians potential biomarkers warranting further analysis for IPMN stratification and treatment targets for invasive PDAC. Future studies should focus on validating these DEGs and pathways in a larger cohort with different grades and biological subtypes of IPMN.

## Author Contributions

**William A. Ziaziaris:** formal analysis (equal), writing – original draft (equal). **Christopher S. H. Lim:** conceptualization (equal), formal analysis (equal). **Loretta Sioson:** methodology (equal), writing – review and editing (equal). **Anthony J. Gill:** supervision (equal), writing – review and editing (equal). **Jaswinder S. Samra:** supervision (equal), writing – review and editing (equal). **Sumit Sahni:** conceptualization (equal), formal analysis (equal), methodology (equal), project administration (equal), writing – review and editing (equal). **Anubhav Mittal:** supervision (equal), writing – review and editing (equal).

## Conflicts of Interest

The authors declare no conflicts of interest.

## Supporting information


Data S1.

## Data Availability

The data supporting the findings of this study are available from the corresponding author upon reasonable request.
