# Supplementary material for: Gene Expression Profiling of Pancreatic Ductal Adenocarcinoma Arising From Intraductal Papillary Mucinous Neoplasms of the Pancreas
Source: Cancer Med. 2024 Dec 11;13(23):e70499. doi: 10.1002/cam4.70499 (PMC11632396; doi:10.1002/cam4.70499)

Table S1: 30 additional genes added to the standard nCounter® PanCancer Progression Panel (©NanoString Technologies Inc, Seattle, WA, USA, 2022)

| **Additional Genes** |
| --- |
| ABCB1 |
| BECN1 |
| CA11 |
| CD274 |
| CD276 |
| CTLA4 |
| DCK |
| EPHA3 |
| FSCN1 |
| HSPA5 |
| IDO1 |
| ITIH4 |
| ITPRIP |
| MAP1LC3B |
| MSLN |
| MUC16 |
| PDCD1LG2 |
| PDE8B |
| PGAM1 |
| PRKAA1 |
| PRKAA2 |
| PTGES2 |
| RACGAP1 |
| RASGRF2 |
| RBP1 |
| S100A2 |
| S100A4 |
| SLC29A1 |
| SQSTM1 |
| TMPO |

Table S2: All 159 significantly up or downregulated genes in the nCounter assay after false discovery rate analysis. Fold change was calculated based on average expression of genes in PDAC versus IPMN. False discovery rate (FDR) correction using the Benjamini and Hochberg was used to determine q-values.

| **Gene** | **Fold Change** | **q-value** | **p-value** | **SignalP** |
| --- | --- | --- | --- | --- |
| S100A2 | 4.569 | 0.038 | 0.023 | 0 |
| MUC16 | 3.704 | 0.011 | 0.004 | 0.0001 |
| CEACAM6 | 3.438 | 0.016 | 0.008 | 0.9988 |
| ELK3 | 2.935 | 0.002 | 0.001 | 0 |
| CLDN4 | 2.844 | 0.005 | 0.002 | 0.0537 |
| CEACAM1 | 2.763 | 0.028 | 0.014 | 0.9997 |
| IL1RN | 2.706 | 0.042 | 0.028 | 0.9997 |
| PLAUR | 2.620 | 0.029 | 0.014 | 0.9998 |
| KRT19 | 2.580 | 0.039 | 0.024 | 0 |
| S100A14 | 2.577 | 0.047 | 0.035 | 0 |
| TNFRSF12A | 2.475 | 0.005 | 0.001 | 0.9998 |
| POSTN | 2.461 | 0.018 | 0.008 | 0.9997 |
| GREM1 | 2.438 | 0.033 | 0.018 | 0.9998 |
| BGN | 2.396 | 0.026 | 0.013 | 0.9996 |
| ANGPTL2 | 2.321 | 0.029 | 0.015 | 0.9997 |
| HK2 | 2.273 | 0.021 | 0.009 | 0 |
| HOXB3 | 2.259 | 0.027 | 0.013 | 0 |
| CYBB | 2.241 | 0.010 | 0.004 | 0 |
| ANXA2P2 | 2.200 | 0.012 | 0.006 | 0 |
| LGALS1 | 2.140 | 0.006 | 0.002 | 0 |
| ITGA11 | 2.116 | 0.024 | 0.011 | 0.9996 |
| TWIST1 | 2.102 | 0.047 | 0.034 | 0 |
| OAS1 | 2.070 | 0.022 | 0.009 | 0 |
| THBS2 | 2.068 | 0.019 | 0.008 | 0.9997 |
| INHBA | 2.050 | 0.038 | 0.023 | 0.9997 |
| APOE | 2.039 | 0.034 | 0.019 | 0.9993 |
| SLPI | 2.038 | 0.022 | 0.009 | 0.9998 |
| DAG1 | 2.027 | 0.002 | 0.001 | 0.9998 |
| CLDN7 | 2.008 | 0.006 | 0.002 | 0.1194 |
| ITGB6 | 2.001 | 0.039 | 0.024 | 0.9998 |
| TMPRSS4 | 1.997 | 0.025 | 0.011 | 0 |
| OLFML2B | 1.964 | 0.007 | 0.002 | 0.8815 |
| PYCARD | 1.964 | 0.037 | 0.022 | 0 |
| PTTG1 | 1.933 | 0.031 | 0.015 | 0 |
| PKM | 1.925 | 0.004 | 0.001 | 0 |
| CLEC2B | 1.917 | 0.014 | 0.007 | 0.0005 |
| STAT1 | 1.908 | 0.004 | 0.001 | 0 |
| ALDOA | 1.908 | 0.029 | 0.015 | 0 |
| FSCN1 | 1.883 | 0.023 | 0.011 | 0 |
| LY96 | 1.865 | 0.041 | 0.026 | 0.8056 |
| ENO1 | 1.864 | 0.026 | 0.013 | 0 |
| LDHA | 1.836 | 0.024 | 0.011 | 0 |
| ITGA3 | 1.825 | 0.036 | 0.021 | 0.9977 |
| TGFBI | 1.822 | 0.044 | 0.028 | 0.9998 |
| TPM2 | 1.812 | 0.050 | 0.038 | 0 |
| RHOA | 1.806 | 0.028 | 0.013 | 0 |
| LAMA4 | 1.792 | 0.043 | 0.028 | 0.9971 |
| TACSTD2 | 1.778 | 0.008 | 0.002 | 0.9998 |
| ISLR | 1.733 | 0.021 | 0.009 | 0.9998 |
| SNAI2 | 1.726 | 0.019 | 0.008 | 0 |
| COL5A1 | 1.713 | 0.039 | 0.024 | 0.9992 |
| COL1A2 | 1.708 | 0.030 | 0.015 | 0.9989 |
| TGFB1 | 1.687 | 0.003 | 0.001 | 0.9997 |
| CRIP2 | 1.678 | 0.045 | 0.033 | 0 |
| PPP3R1 | 1.632 | 0.003 | 0.001 | 0 |
| CD276 | 1.601 | 0.011 | 0.005 | 0.9996 |
| RRAS | 1.599 | 0.038 | 0.023 | 0 |
| PGAM1 | 1.578 | 0.017 | 0.008 | 0 |
| CDK14 | 1.544 | 0.035 | 0.021 | 0 |
| SP1 | 1.541 | 0.014 | 0.006 | 0 |
| RUNX1 | 1.529 | 0.018 | 0.008 | 0 |
| DPYSL3 | 1.515 | 0.023 | 0.011 | 0 |
| GPI | 1.498 | 0.030 | 0.015 | 0.9997 |
| MTMR14 | 1.489 | 0.008 | 0.002 | 0 |
| PGK1 | 1.483 | 0.049 | 0.038 | 0 |
| PLXNC1 | 1.468 | 0.035 | 0.019 | 0.6964 |
| NME1 | 1.454 | 0.048 | 0.038 | 0 |
| WIPF1 | 1.452 | 0.028 | 0.013 | 0 |
| TIMP2 | 1.449 | 0.049 | 0.038 | 0.9998 |
| PDCD10 | 1.446 | 0.009 | 0.003 | 0 |
| MAP1LC3B | 1.428 | 0.027 | 0.013 | 0 |
| BECN1 | 1.422 | 0.033 | 0.018 | 0 |
| RB1 | 1.409 | 0.012 | 0.005 | 0 |
| CDC42 | 1.395 | 0.016 | 0.008 | 0 |
| CEP170 | 1.351 | 0.047 | 0.035 | 0 |
| BRMS1 | 1.350 | 0.013 | 0.006 | 0 |
| PLEKHO1 | 1.311 | 0.049 | 0.038 | 0 |
| SNRPF | 1.308 | 0.034 | 0.018 | 0 |
| EIF4E2 | 1.283 | 0.042 | 0.028 | 0 |
| RBX1 | 1.272 | 0.007 | 0.002 | 0 |
| DENR | 1.267 | 0.016 | 0.008 | 0 |
| PIK3CA | 1.256 | 0.034 | 0.018 | 0 |
| SKP1 | 1.249 | 0.031 | 0.015 | 0 |
| EGLN2 | 1.248 | 0.045 | 0.033 | 0 |
| VEGFB | 1.218 | 0.046 | 0.033 | 0.9997 |
| RAF1 | 1.214 | 0.043 | 0.028 | 0 |
| DNAJC14 | 1.198 | 0.048 | 0.035 | 0 |
| ERBB2IP | 1.188 | 0.045 | 0.033 | 0 |
| PKN1 | 1.185 | 0.015 | 0.007 | 0 |
| AKT2 | 1.165 | 0.048 | 0.038 | 0 |
| UBA52 | 1.149 | 0.046 | 0.033 | 0 |
| S100A7 | -1.166 | 0.046 | 0.033 | 0 |
| SMAD4 | -1.213 | 0.031 | 0.015 | 0 |
| TMUB2 | -1.257 | 0.026 | 0.012 | 0.0001 |
| VIT | -1.271 | 0.032 | 0.015 | 0.9997 |
| KRIT1 | -1.282 | 0.036 | 0.021 | 0 |
| SLC29A1 | -1.301 | 0.036 | 0.021 | 0.0004 |
| RBL2 | -1.313 | 0.018 | 0.008 | 0 |
| PLA2G2A | -1.327 | 0.018 | 0.008 | 0.9997 |
| MED23 | -1.338 | 0.040 | 0.024 | 0 |
| VPS13A | -1.349 | 0.028 | 0.013 | 0 |
| ITGA8 | -1.354 | 0.043 | 0.028 | 0.9822 |
| EIF2AK3 | -1.420 | 0.033 | 0.018 | 0.7206 |
| ACVR1C | -1.426 | 0.000 | 0.001 | 0.9997 |
| PECAM1 | -1.433 | 0.043 | 0.028 | 0.9998 |
| EPAS1 | -1.463 | 0.017 | 0.008 | 0 |
| CD34 | -1.496 | 0.032 | 0.017 | 0.9992 |
| ERMP1 | -1.527 | 0.035 | 0.021 | 0 |
| RAMP2 | -1.538 | 0.044 | 0.028 | 0.9994 |
| MMP3 | -1.571 | 0.013 | 0.006 | 0.9998 |
| SPARCL1 | -1.579 | 0.037 | 0.021 | 0.9994 |
| PPP1R16B | -1.583 | 0.032 | 0.017 | 0 |
| NPR1 | -1.584 | 0.021 | 0.009 | 0.9988 |
| HIPK2 | -1.617 | 0.015 | 0.007 | 0 |
| KCNJ8 | -1.636 | 0.033 | 0.018 | 0 |
| MMRN2 | -1.643 | 0.044 | 0.03 | 0.4503 |
| HSPA5 | -1.647 | 0.015 | 0.007 | 0.9997 |
| GSN | -1.670 | 0.042 | 0.028 | 0.9997 |
| S1PR1 | -1.691 | 0.013 | 0.006 | 0.0002 |
| TAL1 | -1.702 | 0.011 | 0.004 | 0.0001 |
| JAM2 | -1.710 | 0.003 | 0.001 | 0.9997 |
| PTPRB | -1.727 | 0.024 | 0.011 | 0.9998 |
| TEK | -1.763 | 0.019 | 0.008 | 0.9998 |
| PROK2 | -1.771 | 0.041 | 0.026 | 0.9998 |
| CD24 | -1.776 | 0.041 | 0.028 | 0.9997 |
| TNXB | -1.820 | 0.020 | 0.008 | 0.9998 |
| AQP1 | -1.824 | 0.010 | 0.004 | 0 |
| SERPINF1 | -1.832 | 0.040 | 0.024 | 0.9996 |
| GALNT7 | -1.916 | 0.020 | 0.009 | 0 |
| CXCL12 | -1.973 | 0.030 | 0.015 | 0 |
| SOX17 | -1.997 | 0.022 | 0.009 | 0 |
| CXCL17 | -2.001 | 0.048 | 0.038 | 0.9998 |
| CHRNA7 | -2.010 | 0.009 | 0.003 | 0.9997 |
| CLEC3B | -2.023 | 0.025 | 0.012 | 0.9998 |
| EMCN | -2.041 | 0.012 | 0.005 | 0.9997 |
| CHAD | -2.049 | 0.013 | 0.006 | 0.9998 |
| NCAM1 | -2.070 | 0.006 | 0.002 | 0.9997 |
| KDR | -2.133 | 0.009 | 0.003 | 0.9997 |
| EPHB3 | -2.165 | 0.014 | 0.007 | 0.9987 |
| SFRP1 | -2.209 | 0.023 | 0.01 | 0.9998 |
| ADAMTS1 | -2.222 | 0.005 | 0.002 | 0.3637 |
| ITM2A | -2.343 | 0.010 | 0.004 | 0.0003 |
| FAM174B | -2.440 | 0.002 | 0.001 | 0.0003 |
| CD36 | -2.456 | 0.001 | 0.001 | 0 |
| LIFR | -2.478 | 0.004 | 0.001 | 0.8354 |
| IGF1 | -2.488 | 0.017 | 0.008 | 0.9488 |
| FBLN1 | -2.490 | 0.003 | 0.001 | 0.9997 |
| SV2B | -2.515 | 0.037 | 0.021 | 0 |
| DPT | -2.577 | 0.020 | 0.009 | 0.9998 |
| ADAM28 | -2.608 | 0.023 | 0.011 | 0.9985 |
| STAB1 | -2.619 | 0.008 | 0.002 | 0.9998 |
| SLC44A4 | -2.638 | 0.008 | 0.002 | 0 |
| ITIH4 | -2.734 | 0.027 | 0.013 | 0.9998 |
| TFPI2 | -2.914 | 0.038 | 0.023 | 0.9998 |
| OGN | -3.016 | 0.007 | 0.002 | 0.9998 |
| CLU | -3.385 | 0.001 | 0.001 | 0 |
| CHRDL1 | -4.274 | 0.001 | 0.001 | 0.9997 |
| PTX3 | -5.358 | 0.025 | 0.011 | 0.9998 |
| ALB | -27.158 | 0.040 | 0.026 | 0.9997 |

Table S3: Upstream regulator analysis.

| **Upstream Regulator** | **Expr Fold Change** | **Molecule Type** | **Predicted Activation State** | **Activation z-score** | **p-value of overlap** |
| --- | --- | --- | --- | --- | --- |
| TGFB1 | 1.687 | Growth Factor | Activated | 4.641 | 1.65E-31 |
| POU5F1 |  | Transcription Regulator | Activated | 2.909 | 5.02E-09 |
| AGT |  | Growth Factor | Activated | 2.861 | 5.57E-11 |
| ESR2 |  | Ligand-Dependent Nuclear Receptor | Activated | 2.642 | 2.18E-16 |
| Interferon alpha |  | Group | Activated | 2.529 | 0.000000468 |
| NQO1 |  | Enzyme | Activated | 2.408 | 0.000000177 |
| STAT1 | 1.908 | Transcription Regulator | Activated | 2.401 | 0.000165 |
| KLF6 |  | Transcription Regulator | Activated | 2.375 | 0.00000515 |
| HIF1A |  | Transcription Regulator | Activated | 2.333 | 1.08E-17 |
| SMAD3 |  | Transcription Regulator | Activated | 2.326 | 3.89E-11 |
| Tgf beta |  | Group | Activated | 2.239 | 6.37E-15 |
| HMGA1 |  | Transcription Regulator | Activated | 2.236 | 0.00173 |
| USF1 |  | Transcription Regulator | Activated | 2.236 | 0.0000633 |
| PSME3 |  | Peptidase | Activated | 2.236 | 0.000000011 |
| CD38 |  | Enzyme | Activated | 2.236 | 0.00108 |
| CSF2 |  | Cytokine | Activated | 2.23 | 2.53E-08 |
| SLC2A3 |  | Transporter | Activated | 2.224 | 7.82E-09 |
| ARNT |  | Transcription Regulator | Activated | 2.219 | 0.00105 |
| HBEGF |  | Growth Factor | Activated | 2.219 | 0.00000766 |
| TWIST2 |  | Transcription Regulator | Activated | 2.213 | 9.74E-08 |
| NFATC2 |  | Transcription Regulator | Activated | 2.2 | 0.00281 |
| PCGEM1 |  | Other | Activated | 2.197 | 0.00000138 |
| BMP2 |  | Growth Factor | Activated | 2.194 | 0.000655 |
| IFN Beta |  | Group | Activated | 2.192 | 0.00103 |
| YAP1 |  | Transcription Regulator | Activated | 2.158 | 0.00000125 |
| SPP1 |  | Cytokine | Activated | 2.157 | 0.000000404 |
| HSF1 |  | Transcription Regulator | Activated | 2.157 | 0.000000036 |
| IL33 |  | Cytokine | Activated | 2.154 | 0.00369 |
| MYC |  | Transcription Regulator | Activated | 2.038 | 4.79E-14 |
| PDGF BB |  | Complex | Activated | 2.03 | 0.0000365 |
| CENPN |  | Other | Activated | 2 | 0.000000029 |
| PALMD |  | Other | Activated | 2 | 0.00000582 |
| THBS2 | 2.068 | other | Activated | 2 | 0.000000406 |
| TNFSF13B |  | Cytokine | Activated | 2 | 0.00498 |
| KAT2A |  | Enzyme | Activated | 2 | 0.00266 |
| SKIC2 |  | Enzyme | Inhibited | -2 | 0.0000542 |
| NEUROG1 |  | Transcription Regulator | Inhibited | -2 | 0.00023 |
| SPRY2 |  | Other | Inhibited | -2 | 0.00167 |
| CR1L |  | Transmembrane Receptor | Inhibited | -2 | 0.0000155 |
| FAS |  | Transmembrane Receptor | Inhibited | -2 | 0.0018 |
| COMMD1 |  | Transporter | Inhibited | -2 | 0.000000092 |
| SP110 |  | Transcription Regulator | Inhibited | -2 | 0.0042 |
| PGR |  | Ligand-Dependent Nuclear Receptor | Inhibited | -2.12 | 6.92E-09 |
| FLCN |  | Other | Inhibited | -2.157 | 0.000000198 |
| SPDEF |  | Transcription Regulator | Inhibited | -2.2 | 5.46E-08 |
| FOXA1 |  | Transcription Regulator | Inhibited | -2.208 | 0.000155 |
| SRF |  | Transcription Regulator | Inhibited | -2.213 | 0.00709 |
| RORC |  | Ligand-Dependent Nuclear Receptor | Inhibited | -2.412 | 0.00000392 |
| OVOL2 |  | Transcription Regulator | Inhibited | -2.449 | 0.000000066 |
| GLI1 |  | Transcription Regulator | Inhibited | -3.461 | 4.12E-09 |

Figure S1: Pathway interactome


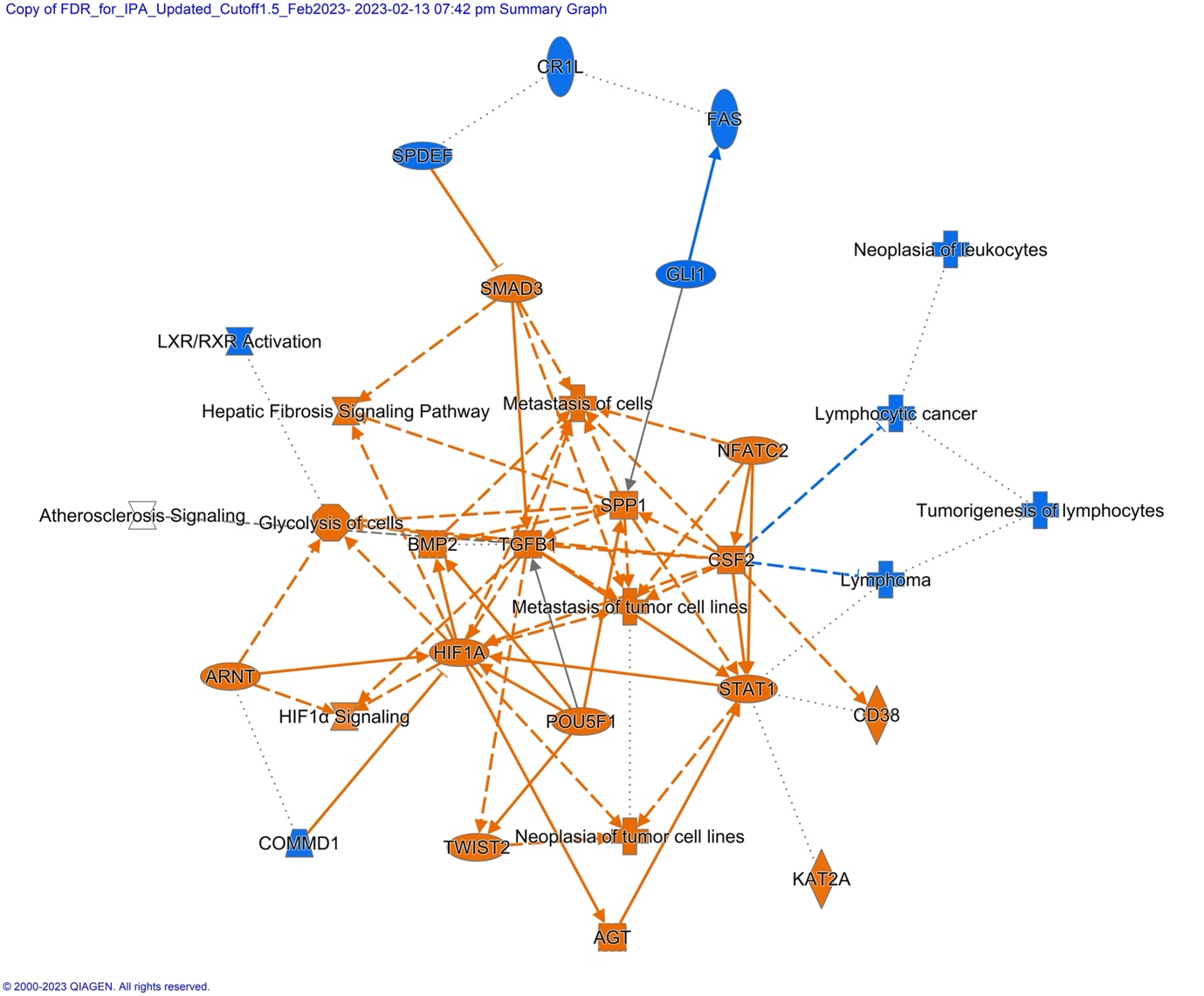

Supplement: Supplementary file 1 — Data S1. [file CAM4-13-e70499-s001.docx]
